# Isolation and Identification of Anthocyanin Component in the Fruits of *Acanthopanax Sessiliflorus* (Rupr. & Maxim.) Seem. by Means of High Speed Counter Current Chromatography and Evaluation of Its Antioxidant Activity

**DOI:** 10.3390/molecules25081781

**Published:** 2020-04-13

**Authors:** Liang Chen, Xiulan Xin, Hui Feng, Shuangshi Li, Qiguang Cao, Xinying Wang, Frank Vriesekoop

**Affiliations:** 1 College of Bioengineering, Beijing Polytechnic, Beijing 100176, China; chenliang@bpi.edu.cn (L.C.); fenghui@bpi.edu.cn (H.F.); lishuangshi@bpi.edu.cn (S.L.); caoqiguang@bpi.edu.cn (Q.C.); 2 MSD R&D (China) Ltd. Co., Beijing 100029, China; wangxinying1986@gmail.com; 3 Department of Food Technology and Innovation, Harper Adams University, Newport TF10 8NB, UK

**Keywords:** anthocyanin, *Acanthopanax sessiliflorus* (Rupr. & Maxim.) Seem., high-speed counter-current chromatography (HSCCC), antioxidant activity

## Abstract

*Acanthopanax sessiliflorus* (Rupr. & Maxim.) Seem. (*Araliaceae*) is one of the most abundant species of genus *Acanthopanax*. The fruits of *A. sessiliflorus* are used in traditional medical protocols as an analgesic, tonic, antidiabetic, antihypertensive, anti-inflammatory, antitumor, and immune-stimulating agent. In this work, we carried out a comprehensive investigation into the anthocyanin components in the fruits of *A. sessiliflorus*. The anthocyanin content in the fresh fruits of *A. sessiliflorus* was determined by high performance liquid chromatography-diode array detection (HPLC/DAD), and the anthocyanin component was isolated from these using high-speed counter-current chromatography (HSCCC) and elucidated by electro-spray ionization-mass spectrometry (ESI/MS), ^1^H- and ^13^C-NMR. Its antioxidant activity was evaluated by ferric-reducing antioxidant power (FRAP) and 2,2-diphenyl-1-picrylhydrazyl (DPPH). We found that *A. sessiliflorus* contained a gross anthocyanin content of 121.35 mg/100 g. HSCCC was successfully used for separation and purification of the primary anthocyanin component, cyanidin 3-xylosyl-galactoside. The antioxidant and radical scavenging tests indicated that cyanidin 3-xylosyl-galactoside is a potent antioxidant.

## 1. Introduction

**Figure 1:**
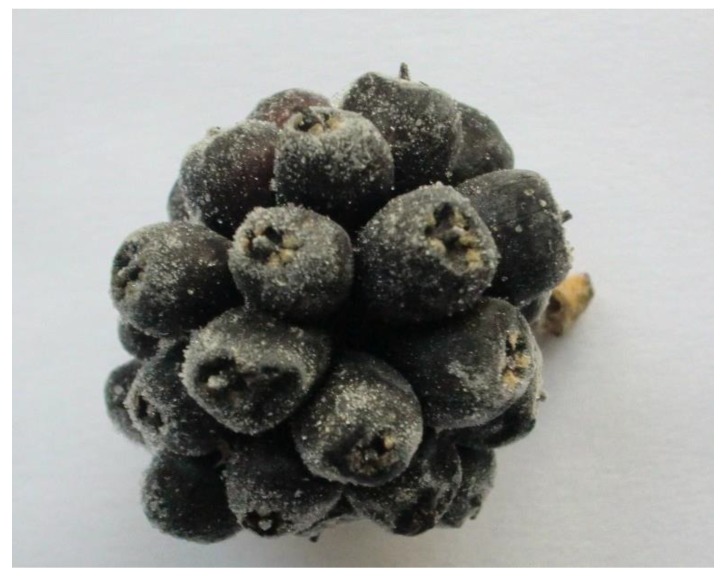
Fruits from *Acanthopanax sessiliflorus* (Rupr. & Maxim.) Seem.

*Acanthopanax sessiliflorus* (Rupr. & Maxim.) Seem. (*Araliaceae*) (Figure 1) is one of the most abundant species of genus *Acanthopanax*, which is a perennial plant native to the northeast region of China, Japan, and Korea. The roots, stems, and leaves of *A. sessiliflorus* elicit a range of biological functions, including antipancreatic lipase, antiplatelet aggregation, and anti-inflammatory effects [1,2,3,4]. Various parts from the plant are used in a wide range of traditional medical applications, including the roots, shoots, bark, leaves, and fruits [2,5,6,7,8,9,10]. The fruits of *A. sessiliflorus* are used in traditional medical protocols as an analgesic, tonic, antidiabetic, antihypertensive, anti-inflammatory, antitumor and immune-stimulating agent [5,6,7].

Many different compounds were extracted and identified from the dried materials of *A. sessiliflorus* fruits for their biological functions, identified as seopoletin, isofraxidin, hyperin, scoparone, protocatechuic acid, ursolic acid, oleanic acid, chiisanogenin, sesamin, daucosterol, β-sitosterol, etc. [11,12,13,14,15]. While most of the above-mentioned compounds provide potential health benefits in terms of analgesic, anticoagulant, or anti-inflammatory actions, only very few antioxidant compounds were identified. An unidentified compound (AE-7-CII) from *A. sessiliflorus* possessing an antioxidative profile and characterized as both an O^2−^ inhibitor and scavenger was described [16]. Up to now, few reports exist regarding anthocyanin analysis in fresh *A. sessiliflorus* fruits. Anthocyanins provide the skins of fruits with colors varying from purple to black and possess a multitude of biological properties, e.g., antidiabetic, anti-inflammatory and antitumor properties [17].

High-speed counter-current chromatography (HSCCC) is an innovative technique used for scale-up separation with minimum sample preparation and permits both normal- and reversed-phase operation [18]. HSCCC is a support-free liquid–liquid partition chromatographic technique shown to be a useful tool for the purification of compounds from natural products [19,20,21,22,23]. As such, HSCCC has some major advantages over more traditional liquid–solid chromatography by eliminating the various complications caused by the solid support matrix, such as adsorptive loss and deactivation of samples, tailing of solute peaks, contamination, etc. It is a relatively simple (low-pressure method) technology and has a relatively low cost of operation due to its use of technical-grade solvents [24]. Finally, it has an excellent yield capacity with a phenomenal sample recovery.

In this study, the anthocyanin content in the fresh fruits of *A. sessiliflorus* was determined by HPLC/DAD, and the anthocyanin component was isolated from these using HSCCC and elucidated by ESI/MS, ^1^H- and ^13^C-NMR. Antioxidant activity was evaluated by 2,2-diphenyl-1-picrylhydrazyl (DPPH) and ferric-reducing antioxidant power (FRAP).

## 2. Results and Discussion

### 2.1. Total Anthocyanin Determination

**Figure 2:**
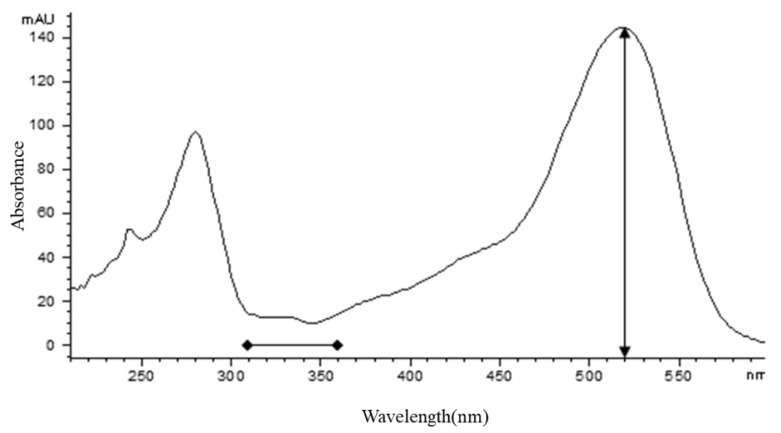
UV-Vis spectrum of the primary peak. The maximum absorbance at 520 nm. The lack of absorbance in the region between 310 and 360 nm (horizontal bar) indicates that the compound might not be acylated.

The gross anthocyanin content was 121.35 mg/100 g, expressed as milligrams of cyanidin 3-glucoside per 100 g of fresh weight by HPLC-DAD. The primary peak (Figure 2) occupied 95.4% of the total peak area and other anthocyanin components were present at trace levels only. Based on the UV-Vis spectrum of the primary peak, the maximum absorbance was 520 nm, which is typical of anthocyanins [25,26]. The absorbance between 310 and 360 nm (Figure 2) suggested that the compound might not be acylated [27]. The absorbance ratio at 440 nm to the absorbance of the visible maximum wavelength for the primary peak (A_440 nm_/A_λmax_ ratio) was found to be 30.08%, indicating that this compound is very likely glycosylated at the C-3 position of the C-ring [28,29].

### 2.2. Selection of Two-Phase Solvent System for Purification

A suitable two-phase solvent system is essential for successful separation by HSCCC. Nonpolar solvents are favored for the isolation of nonpolar target compounds, while polar solvent systems are favored for the isolation of polar compounds. In order to realize efficient separation of the typically hydrophilic anthocyanin compounds, a system of polar solvents was examined. Separation by HSCCC is expressed as its partition coefficient (K), which is usually between 0.4 and 2.5 [20]. A smaller K-value elutes the solute closer to the solvent front at a reduced resolution, while a larger K value tends to yield a better resolution but with broader, more dilute peaks due to a longer elution time [20].

The biphasic solvent system used in this study was composed of methyl tert-butyl ether/*n*-butanol/acetonitrile/water/trifluoroacetic acid, which is commonly used for the separation of anthocyanins [30,31,32]. Since the volume ratio of methyl tert-butyl ether and *n*-butanol has a marked influence on the polarity differential of the two phases, the optimal solvent system was optimized by changing the volume ratio of methyl tert-butyl ether and *n*-butanol. The results demonstrated that methyl tert-butyl ether/*n*-butanol/acetonitrile/water/trifluoroacetic acid (3.5:0.5:1:5:0.01, *v*/*v*) was suitable for separation of the main anthocyanin components from crude *A. sessiliflorus* anthocyanins (CAA).

### 2.3. HSCCC Separation

**Figure 3:**
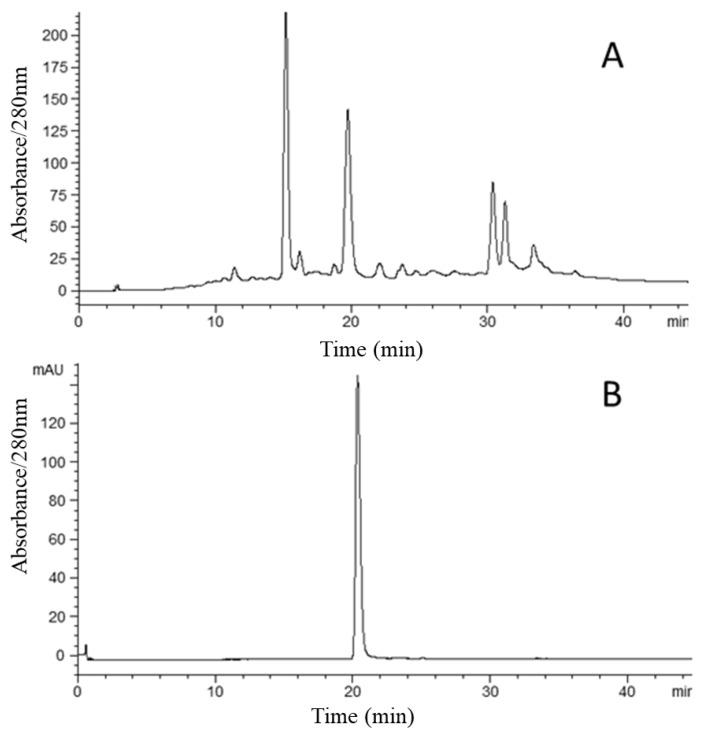
HPLC chromatograms at 280 nm. (**A**) Chromatogram of the *Acanthopanax sessiliflorus* acidified methanol extract; (**B**) cyanidin 3-xylosyl-galactoside from *Acanthopanax sessiliflorus* following purification by means of high-speed counter-current chromatography (HSCCC) (Figure 4).

**Figure 4:**
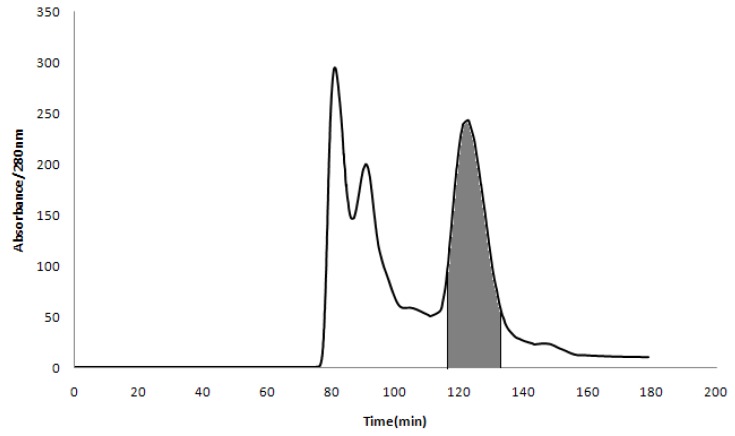
HSCCC chromatogram of the extract from *Acanthopanax sessiliflorus* (Rupr. & Maxim.) Seem. The shaded peak represents the fraction of cyanidin 3-xylosyl-galactoside.

The HPLC detection of CAA at 280 nm indicated that it contained several compounds (Figure 3). While no attempts were made to identify all peaks in Figure 3A, the peak that eluded at approximately 20 min was subsequently identified as cyanidin 3-xylosyl-galactoside (see Figure 3B and Figure 4). The relative concentration of anthocyanin compounds in the crude sample was 13.9%. Figure 4 shows the result obtained from 100 mg of the crude sample by semi-preparative HSCCC. The compounds that eluded between 80 and 90 min, as seen in Figure 4, possessed no color and were therefore not considered to be anthocyanins. The eluent associated with the shaded part of the peak was collected and concentrated. This separation yielded 13.1 mg of anthocyanins at 96.1% purity based on HPLC analysis, with a mass recovery greater than 90%.

### 2.4. Confirmation of Anthocyanin Compound

To confirm the chemical structure of the anthocyanin compound, the fraction purified by HSCCC was subjected to both MS and NMR analyses.

**Figure A1:**
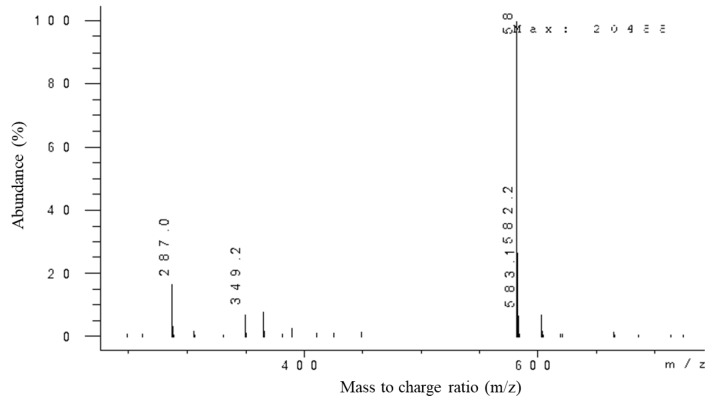
The electro-spray ionization (ESI) mass spectrum obtained from the HSCCC purified fraction.

The anthocyanin fraction shown as the greyed out area in Figure 4 was structurally analyzed by ESI-MS. A clear mass spectrum was obtained where an ion at [M + H]^+^ *m*/*z* 583 indicated the molecular weight of the entire molecule (Figure A1). Based on the *m*/*z* and fragmentation pattern in this study and similar analysis from other studies [25], the anthocyanin was tentatively labeled as cyanidin 3-xylosyl-galactoside. As the fragmentor voltage was increased, a decrease in the relative intensity of the putative cyanidin 3-xylosyl-galactoside ion was observed (data not shown). However, the increase in the fragmentor voltage did result in an increase in a mass ion of *m*/*z* 287, corresponding to the core cyanidin structure of anthocyanidin [33,34]. This positively charged aglycone was most likely formed by the loss of the putative disaccharide moiety (xylose and galactose) from Cya-3-Xyl-Gal.

**Figure 5:**
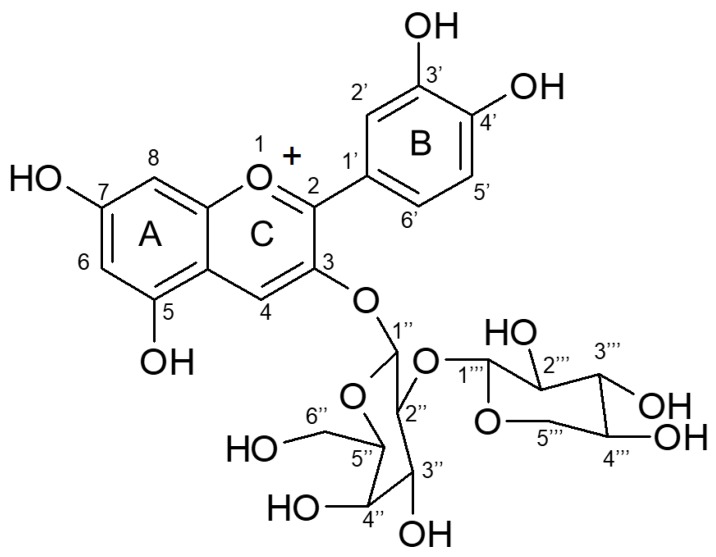
Chemical structure of cyanidin 3-xylosyl-galactoside based on ESI-MS, ^13^C, and ^1^H analyses.

**Figure A2:**
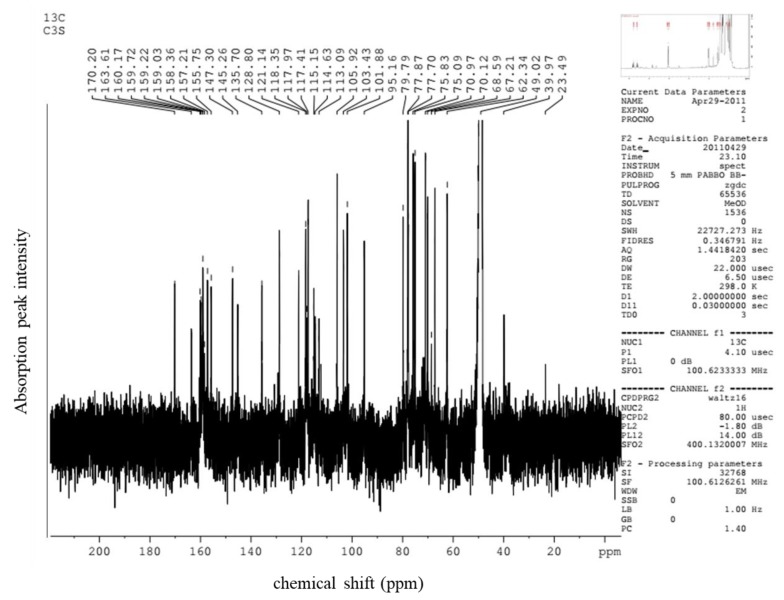
^13^C spectral data (ppm) for the HSCCC-purified cyanidin 3-xylosyl-galactoside.

**Figure A3:**
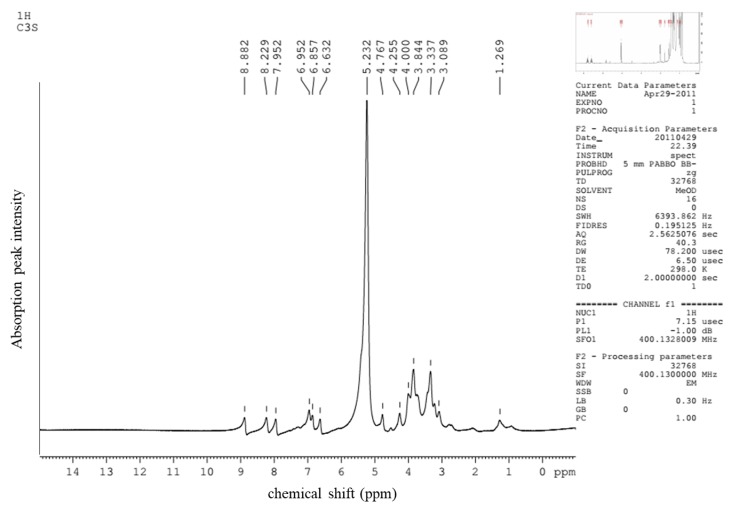
^1^H spectral data (ppm) for the HSCCC-purified cyanidin 3-xylosyl-galactoside.

In order to determine the sugar moiety and the linkage patterns between the cyanidin core and the sugar groups, the purified primary anthocyanin (Figure 4) was further characterized by ^1^H- and ^13^C-NMR analyses (Table 1 and Figure A2 and Figure A3). With regard to the ^1^H-NMR analysis, the compound showed a singlet at 8.88 and doublets at 6.63 and 6.95 for the flavone aromatic protons 4, 6, and 8, respectively. The aromatic protons 2′, 5′, and 6′ were seen as two doublets and a double doublet at 7.95, 6.86, and 8.23, respectively, with *J* values of 2.5 and 9 Hz. The anomeric protons associated with the sugars at 5.39 and 4.71 appeared as doublets with a *J* value of 7.5 Hz, suggesting a diglycoside moiety with β-configuration at the anomeric positions. Other protons corresponding to the diglycoside group appeared as three triplets at 4.26, 3.79, and 3.03, consistent with one, two, and two protons, respectively. The spectrum also contained two doublets at 3.98 and 3.84, along with three double doublets at 3.91, 3.17, and 3.64. A multiplet was seen in the spectrum at 3.35. Many anthocyanins contain glucose within the sugar moiety [25,26]. In our data analysis we favored galactose as the hexose within the diglycoside because of the typical chemical shifts and coupling constants at 2″, 3″, and 4″ in Cya-3-Xyl-Gal [35,36] compared to the typical chemical shifts and coupling constants at 2″, 3″, and 4″ associated with glucose in Cya-3-Xyl-Glu [29]. Furthermore, our ^13^C-NMR spectral data revealed 15 carbons associated with the cyanidin aglycone portion and 11 carbons associated with the sugar moiety. The ^13^C-NMR shifts correlatde very well with the ^1^H-NMR data, especially in regard to the aromatic and carbohydrate carbon observations. The molecular mass of *m*/*z* 583 alongside the observed NMR spectral data corresponded very closely to previously reported results [29,37,38]. As a result of the combined MS and NMR data, the purified fraction was upheld to be cyanidin 3-xylosyl-galactoside (Figure 5).

In addition, the molar absorptivity (ε) of Cya-3-Xyl-Gal (MW = 583) was determined and calculated to be 16,900 L/(mol·cm) from the slope of the plot between λ_max_ (530 nm) over a concentration range of 0–0.05 mmol/L.

Cya-3-Xyl-Gal was previously reported in *A. sessiliflorus* [25], the berries of the Angelica tree (*Aralia elata*) and Spikenard (*Aralia cordata*) [39], and black carrot (*Daucus carota* L.) [40,41]. In black carrot, Cya-3-Xyl-Gal was typically the second most abundant anthocyanin, while it appeared to be the dominant anthocyanin in the berries [25,39].

### 2.5. Antioxidant Activity of Cyanidin 3-Xylosyl-Galactoside

The antioxidant activity of Cya-3-Xyl-Gal was investigated and compared to an equimolar ascorbic acid control using both 2,2-diphenyl-1-picrylhydrazyl (DPPH) and ferric-reducing antioxidant power (FRAP).

**Figure 6:**
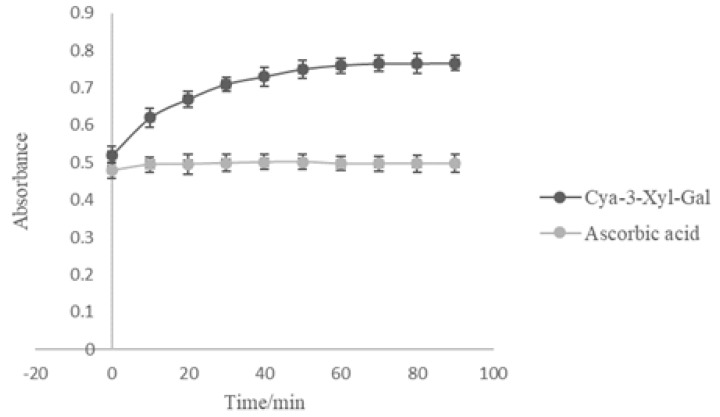
The aging curve of ferric-reducing antioxidant power at 0.1 mmol/L (ascorbic acid and Cya-3-Xyl-Gal).

The FRAP assay measures the reduction of ferric iron (Fe^3+^) to ferrous iron (Fe^2+^) due to the presence of antioxidants and is commonly employed for the routine analysis of single antioxidants and total antioxidant activity of plant extracts [42,43]. Figure 6 presents the results of the aging curve of the ferric-reducing antioxidant power of Cya-3-Xyl-Gal and ascorbic acid at 0.1 mmol/L. After 40 min the ferric-reducing reaction reached equilibrium, resulting in FRAP values of the tested Cya-3-Xyl-Gal and standard ascorbic acid of 234.5 μmol/L and 368.0 μmol/L, respectively. Cya-3-Xyl-Gal showed higher ferric-reducing power than the equimolar ascorbic acid control.

**Figure 7:**
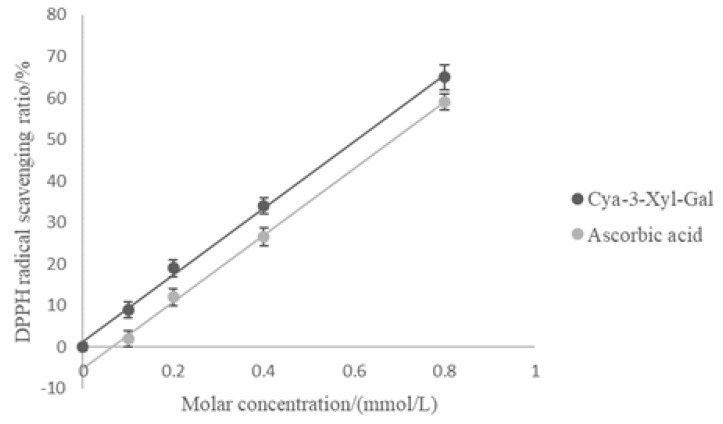
Relationship between the molar concentration of ascorbic acid and Cya-3-Xyl-Gal and DPPH radical scavenging exhaustion for the determination of the IC_50_ values.

The DPPH method is often used to reliably evaluate the free radical scavenging activity of a plant extraction or antioxidant activity due to its stable character [43]. DPPH radical scavenging activity increased with increasing molar concentrations of ascorbic acid and Cya-3-Xyl-Gal (Figure 7). The IC_50_ values (the antioxidant concentration at which 50% of the radical scavenging activity was achieved) of Cya-3-Xyl-Gal and ascorbic acid were 0.63 mmol/L and 0.72 mmol/L, respectively, indicating that the DPPH radical scavenging activity of Cya-3-Xyl-Gal was also greater than that of ascorbic acid.

Both antioxidant activity assay methods revealed Cya-3-Xyl-Gal had higher antioxidant activity compared to an equimolar ascorbic acid preparation, implying that Cya-3-Xyl-Gal may be a potent antioxidant.

## 3. Materials and Methods

### 3.1. Chemicals and Reagents

The fully mature fruits of *Acanthopanax sessiliflorus* (Rupr. & Maxim.) Seem. were handpicked from all sides from a number of representative plants in Jilin Province in Northeast China during the period of August to October and stored at −40 °C as soon as possible for subsequent chemical analysis. These samples were authenticated by Prof. Liming Zhang at Ningxia Medical University and a voucher specimen (No. Y201107001) deposited at the College of Bioengineering, Beijing Polytechnic.

All organic solvents used were of analytical grade and obtained from Beijing Chemical Factory (Beijing, China). The methanol used for HPLC analysis was of chromatography grade and obtained from Honeywell B&J (Beijing, China). The standard for cyanidin was obtained from Mansite biological technology Co., Ltd. (Chengdu, China). AB-8 macroporous resin was obtained from Baoen adsorbent material Technology Co., Ltd. (Cangzhou, China).

### 3.2. Instruments and Apparatus

The semi-preparative HSCCC instrument used in this study was a TBE-300A high-speed countercurrent chromatograph (Shanghai Tauto Biotech Co., Ltd., Shanghai, China), which was fitted out and operated as described previously [32]. An HX1050 constant-temperature circulating instrument (Beijing Boyikang Lab Instrument, Beijing, China) was used to maintain a constant separation temperature. A model TBP-50A constant flow pump (Shanghai Tauto Biotech Co., Ltd., Shanghai, China) was employed to supply the solvent into the column, while the eluent was monitored with a model TBD-23UV detector (Shanghai Tauto Biotech Co., Ltd., Shanghai, China).

The analytical HPLC equipment was an Agilent 1100 system [32] fitted with a Zorbax SB-C18 column (250 mm × 4.6 mm, i.d., 5 mm, Agilent Technologies Inc., Shanghai, China).

Electro-spray ionization mass (ESI/MS) analysis and nuclear magnetic resonance (NMR) analysis were carried out by analysts at the Center of Analysis, Beijing University of Chemical Technology, Beijing, China.

### 3.3. Total Anthocyanin Determination

The anthocyanins were thoroughly extracted from three independent 10 g samples of fresh *A. sessiliflorus* fruits with methanol acidified with 0.1% HCl [32]. The extracts were then analyzed by HPLC. A commercially available standard of cyanidin 3-glucoside was used to create a calibration curve, which yielded a calibration equation of Y = 2233 × X + 13.38 (R^2^ = 0.9999). The content was expressed as milligrams of cyanidin per 100 g of fresh weight.

### 3.4. Bulk Anthocyanin Extract of A. sessiliflorus

The fruits of *A. sessiliflorus* (1 kg fresh weight) were extracted with acidified methanol acidified with 0.1% HCl at room temperature without continuous agitation for 24 h. This process was repeated three times, after which the extracts were combined. The ratio of the extraction solvent to fruit was 10:1 (v/w). The combined extract was evaporated to form a syrup of about 200 mL. The syrup was diluted to 500 mL with distilled water acidified with 0.1% HCl. This aqueous solution was subjected to column chromatography (45 mm × 50 cm) using an AB-8 macroporous resin in order to separate the anthocyanins. The anthocyanins were eluted under gravity with 1 L acidified distilled water followed by 2 L acidified ethanol acidified with 0.1% HCl. The ethanol elution solution was first evaporated into a syrup and then freeze-dried to yield crude *A. sessiliflorus* anthocyanins (CAA).

### 3.5. Preparation of Two-Phase Solvent Systems and Sample Solutions

The solvents were mixed in a separation funnel and thoroughly equilibrated by vigorous shaking at room temperature, after which the two phases were allowed to separate. The two phases were subsequently degassed by sonication for 20 min before use. The sample solution for HSCCC was prepared as described previously [32] by dissolving 100 mg of CAA into a 10 mL two-phase solvent system.

### 3.6. Determination of Partition Coefficients (K)

The partition coefficients (K) were determined as described previously [44]. Briefly, a small amount of CAA was dropped into a 10 mL test tube, to which 2 mL of each phase of the equilibrated two-phase solvent system (methyl *tert*-butyl ether/*n*-butanol/acetonitrile/water/trifluoroacetic acid) was added. The tube was shaken vigorously, after which the two phases were allowed to separate. A 1 mL sample of each phase was removed and the solvents were removed by evaporation, after which the residue of each phase was redissolved in acidified methanol. The resulting solutions were analyzed by HPLC, as described in Section 3.3. The K-values were calculated by the ratio between the peak areas.

### 3.7. HSCCC Separation

HSCCC separation was carried out according to the method previously published [32]. Briefly, we applied a head–tail elution mode with the organic phase as the stationary phase. To start with, the multilayer coiled column was completely filled with the organic phase. The aqueous phase was pumped into the head of the column at a flow rate of 2.0 mL/min while running at 850 rpm at 25 °C. The UV detector was set at 280 nm. Samples were injected into the separation column after a hydrodynamic equilibrium was established.

### 3.8. Analytical Controls and Structure Elucidation

Quantitative analysis of the anthocyanins by HPLC were as described previously [32]. UV-Vis absorption spectra were recorded using a photodiode array detector. Spectral measurements were made over the wavelength range 200~600 nm in steps of 2 nm. Anthocyanin components were detected at both 280 nm and 520 nm.

The purified compound was identified by electron impact MS, ^1^H-NMR, and ^13^C-NMR spectrometry carried out by analysts at the Center of Analysis, Beijing University of Chemical Technology. NMR spectra were performed in CD_3_OD/CF_3_COOD (95:5 *v*/*v*) using a Bruker high-resolution AV500NMR spectrometer at 500 MHz (Bruker Bio Spin Corporation, Billerica, MA, USA).

### 3.9. Determination of Molar Absorptivity of Anthocyanin Component

The molar absorptivity (ε) of the anthocyanin component was determined by dissolving a series of known quantities of purified anthocyanin in acidified methanol (0.1% HCl). The absorbance of the anthocyanin in this dilution series was measured in a Cary 50 Bio UV-Visible spectrophotometer (Varian, Inc., Palo Alto, CA, USA) at 530 nm using 1 cm path length quartz cells at λ_max_.

### 3.10. Evaluation of Antioxidant Activity

The antioxidant capacity of ascorbic acid as a standard reference compound and the purified anthocyanin were determined using in vitro FRAP and DPPH antioxidant assays.

The FRAP assay employed 3 aqueous stock solutions, namely, 0.1 mol/L acetate buffer (pH 3.6), 10 mmol/L tripyridyl triazine, and 20 mmol/L ferric chloride, as described previously [45]. Just prior to use, these solutions were combined (10:1:1, *v*/*v*/*v*) to form the FRAP reagent. The FRAP reagent and the samples were mixed thoroughly, after which the absorbance of the reaction mixture was determined at 593 nm using a Cary 50 Bio UV-Visible spectrophotometer (Varian, Inc., Palo Alto, CA, USA). The total antioxidant capacity of anthocyanin samples and ascorbic acid was determined against a ferrous sulphate standard of known FRAP value over a range of 0~500 μM.

The DPPH assay employed a fresh methanolic DPPH solution as described previously [46]. The freshly prepared DPPH solution was transferred into a plastic cuvette and anthocyanin sample, or ascorbic acid was added. The solution was stirred and left to stand in the dark for 30 min, after which the absorbance was measured at 515 nm. The radical scavenging activity was expressed as the percentage of DPPH radical elimination, which was calculated according to the following equation:

R (%) = (A_0_ − A_1_)/A_0_ × 100%,

where R is the radical scavenging activity of a sample, A_0_ is the absorbance of the sample at the start of the reaction, and A_1_ is the absorbance of the sample at the end of the reaction.

## 4. Conclusions

We identified the principle anthocyanin in *Acanthopanax sessiliflorus* (Rupr. & Maxim.) Seem as Cya-3-Xyl-Gal. Confirmation of identity was based on ESI-MS, ^13^C-NMR, and ^1^H-NMR analyses. The gross anthocyanin content of *A. sessiliflorus* was 121.35 mg/100 g. The application of HSCCC allowed for the successful separation and purification of the primary anthocyanin, Cya-3-Xyl-Gal, which was subsequently used for antioxidant and radical scavenging testing. Both the in vitro antioxidant and radical scavenging tests (FRAP and DPPH, respectively) demonstrated that Cya-3-Xyl-Gal isolated from *A. sessiliflorus* possesses potent antioxidant potential.

## Figures and Tables

**Table 1:** ^13^C and ^1^H NMR spectral data (ppm) for cyanidin 3-xylosyl-galactoside.

Cyanidin 3-Xylosyl-Galactoside	^13^C (ppm) Also Figure A2	^1^H (ppm) Also Figure A3
aglycone		
2	164.08	
3	145.33	
4	136.12	8.88 s
5	159.18	
6	103.41	6.63 d2.0
7	170.34	
8	95.00	6.95 d9.5
9	157.47	
10	113.19	
1′	121.25	
2′	118.58	7.96 d2.5
3′	147.29	
4′	155.78	
5′	117.39	6.86 d1.0
6′	128.59	8.23 dd2.5,9.0
galactose		
1″	102.10	5.39 d7.5
2″	80.02	4.26 t9.0
3″	75.10	3.91 dd3.5,9.5
4″	70.06	3.98 d3.0
5″	77.70	3.84 d9.0
6A″	62.33	3.81 d3.5
6B″		3.79 t5.5
xylose		
1‴	106.01	4.71 d7.5
2‴	75.81	3.17 dd8.0,9.0
3‴	77.87	3.35 m
4‴	70.97	3.64 dd5.0,11.50
5‴	67.16	3.73 d2.0
6‴		3.03 t11.0
